# Embedding a Palliative Care Nurse Consultant Within a General Medicine Ward: A Prospective Exploratory Study

**DOI:** 10.1111/jocn.70088

**Published:** 2025-09-01

**Authors:** Jianxia Zhai, Christine Mooney, Olivia Hamilton, Gillian Vesty, Rebecca Millar, Fiona Runacres, Matthew Dellit, Barbora de Courten

**Affiliations:** ^1^ School of Health & Biomedical Science, STEM College RMIT University Melbourne Australia; ^2^ Supportive and Palliative Care Unit Monash Health Melbourne Australia; ^3^ School of Architecture & Urban Design RMIT University Melbourne Australia; ^4^ School of Accounting, Information Systems and Supply Chain, College of Business and Law RMIT University Melbourne Australia; ^5^ Medicine, Nursing and Health Science Department Monash University Melbourne Australia; ^6^ General Medicine Unit and Supportive and Palliative Care Unit Monash Health Melbourne Australia; ^7^ School of Clinical Sciences Monash University Melbourne Australia; ^8^ Department of Diabetes and Vascular Medicine Monash Health Melbourne Australia; ^9^ Department of General Medicine Monash Health Melbourne Australia

**Keywords:** advanced care planning, care coordination, general medicine, internal medicine, multidisciplinary team collaboration, non‐malignant conditions, palliative care

## Abstract

**Aim:**

To describe patient outcomes for patients at high risk of mortality (with a prognosis of three months or less to live) where a Palliative Care Nurse Consultant (PCNC) was embedded in a General Medicine team. To explore patients and/or their carers feedback and allied health, nursing professionals' perspectives on integrating a palliative care approach in the General Medicine ward.

**Design:**

Prospective exploratory study.

**Methods:**

SQUIRE reporting guidelines was adopted for the study reporting. This study was conducted over six weeks in a general medicine ward at Monash Medical Centre in Melbourne, Australia. Participants were 20 patients aged > 65 years with non‐malignant, chronic conditions at high risk of mortality within three months and had 18 nursing and allied health professionals involved in their care. Quantitative data were analysed descriptively and qualitative survey data were analysed thematically.

**Results:**

Twenty patients participated, with an average age of 87 years. 55% spoke a language other than English. PCNC interventions, focused on care coordination and family liaison, were found to facilitate timely referrals to other support services, improve communication and better address end‐of‐life care needs. Healthcare professionals recognised the benefits of PCNC involvement; however, a key qualitative theme was staff reluctance to raise palliative care needs due to perceived role boundaries and limited confidence. While PCNC presence improved communication and advocacy, barriers included time constraints and patient/family resistance.

**Conclusion:**

Embedding a PCNC in a general medicine team appears to enhance care coordination and support timely palliative care integration. Addressing barriers and optimising workflow can improve patient, carer and clinician experience as well as improve resource utilisation.

**Implications for the Profession and/or Patient Care:**

The model has the potential to enhance patient‐centred care and clinician support in acute general medicine settings.

**Impact:**

The research will have an impact on acute care settings, particularly general medicine units, by informing models of integrated palliative care for patients with complex needs and enhancing staff capability and confidence in providing timely, person‐centred care.

**Patient or Public Contribution:**

Patients or members of the public were not involved in the design, conduct, analysis or manuscript preparation of this study. The project was a prospective observational study with limited scope and resources, which did not include a formal patient or public involvement component.

**Summary**

- What does this paper contribute to the wider global clinical community
  ○ Demonstrates a practical model for integrating palliative care into general medicine wards to support older patients with complex, non‐malignant conditions.
  ○ Highlights the role of Palliative Care Nurse Consultants in improving care coordination, communication and early end‐of‐life planning.
  ○ Provides evidence to inform service redesign in acute care settings internationally, promoting timely, person‐centred palliative care.

## 1 Background

Globally, populations are ageing, and the prevalence of multimorbidity is rising, resulting in increased demand for health systems to manage complex, chronic and life‐limiting conditions (Sleeman et al. 2019). Despite this growing need, access to palliative care remains inequitable, particularly among patients with non‐malignant diseases such as advanced heart failure and chronic obstructive pulmonary disease (COPD) (Kingston et al. 2020). Palliative care services continue to be underutilised and are often introduced late in the disease trajectory, even though early integration is associated with improved quality of life, symptom management and healthcare utilisation (Mah et al. 2024; Quinn et al. 2020; Walker et al. 2025).

In Australia, this trend is mirrored by national data. Although Australians are living longer, the ‘years lived in ill health’ have increased, leading to an increase in the utilisation of health care services (Australian Institute of Health and Welfare 2023). Most older Australians are living with chronic, non‐malignant diseases and die experiencing multiple health conditions. In 2022, 191,000 deaths were registered in Australia. It was found that 80% had more than 1 cause of death and almost 25% had 5 or more causes recorded (Australian Institute of Health and Welfare 2023). In addition, the number of causes recorded per death is slowly increasing over time (Australian Institute of Health and Welfare 2023).

Patients admitted to General Medicine (Internal Medicine) ward are typically older, often with multiple comorbidities and associated functional and cognitive decline (Geyskens et al. 2022; Hoyer et al. 2016). Whilst it is acknowledged at a population level that these patients have a high risk of short‐term mortality, how this knowledge is translated into practice is unknown. The extent to which patients themselves understand the life limiting nature of their medical conditions is also unclear. Advanced care planning (ACP) participation within this population remains low, with minimal information provided to patients about their treatment preferences (Frechman et al. 2020). Discharging patients without discussing prognosis or establishing a values‐based future management plan can lead to poor patient outcomes and contribute to inappropriate utilisation of health resources, such as post‐discharge emergency room visits and readmissions. The aim of palliative care is to relieve physical, psychological and emotional suffering; coordinate care across health care settings; contribute to future care planning and support discussions regarding sensitive issues such as end of life care (Milazzo et al. 2020). The need for palliative care support is increasing, with the estimated demand in Australia expected to increase by 50% between now and 2035, and further double by 2050 (Palliative Care Australia 2024). Evidence suggests that proactive palliative care involvement could help to reduce health service utilisation whilst also improving care coordination between patients, families and healthcare professionals both within and beyond hospital boundaries, as well as improving wellbeing and lowering bereavement costs (Chapman et al. 2018; Forbat et al. 2020; Garon et al. 2024; Gatta and Turnbull 2018; Hudson et al. 2010; Kang et al. 2024; Kiyange et al. 2024; Ma et al. 2019; Martin et al. 2024; Petrillo et al. 2024; Walsh et al. 2007). For every dollar spent on palliative care services in an acute care setting in 2019, there was a return on investment of between $1.36 and $2.13 (Palliative care Australia 2024). With varying patient groups being diverted from diverse specialties and clinical settings to access palliative care, there is evidence that earlier palliative care integration into a general medicine ward workflow (before patients are imminently dying) can offer important value‐based benefits for both patients, their families and healthcare services (Aslakson et al. 2014).

Despite this, patients with non‐malignant conditions experience lower referral rates and delayed engagement with palliative care services compared to those with malignant conditions (Hunter 2018; Senderovich and Jimenez Lopez 2021; Velaga et al. 2023). Referrals to palliative care often occur during the terminal phase (within days of death), when patients may no longer be able to communicate their treatment preferences, leaving families to make decisions on their behalf.

In Australia, despite increasing recognition of the need for timely palliative care and benefits for palliative care integration into general medicine ward workflow, there remains a notable gap in how this care is systematically integrated into acute General Medicine (Internal Medicine) wards. The optimal service delivery model has yet to be determined.

Nurse consultants with palliative care expertise are uniquely positioned to bridge this gap. Their clinical autonomy, interdisciplinary communication skills and established presence in acute care settings make them suitable agents for early engagement with patients and families.

## 2 Aims

The primary aim was to describe patient outcomes and feedback where a PCNC was embedded within the General Medicine ward in a tertiary hospital setting for patients with complex healthcare needs and an increased risk of mortality. The secondary aim of the study was to explore the views and practice of allied health and nursing professionals about embedding a palliative care approach in General Medicine wards.

## 3 Methods

### 3.1 Participants and Settings

This prospective observational study was conducted with patients and health care professionals in a General Medicine Ward at Monash Medical Centre in Melbourne, Australia.

Patient participants included patients aged 65 years or older who were receiving treatment for non‐malignant diseases under the care of the General Medicine Ward. Eligibility required identification by the Registrar or Consultant of the treating team as being at high risk of death within the next three months, with limited potential for reversibility of their current clinical condition. Patients already receiving support from Palliative Care services were excluded from the study. Nursing and allied health professionals, with at least six months of experience in the General Medicine units at Monash Medical Centre, and directly involved in patient care, were also included in the study.

The patient participants were recruited using consecutive sampling: all patients admitted to the ward during the study period were screened for eligibility by the PCNC using clinical judgement and validated tools (e.g., the Surprise Question, Palliative performance scale). Of these, 20 patients who met the inclusion criteria were included in the study.

A formal power calculation was not conducted, as this was a prospective observational quality assurance study with a fixed six‐week timeframe. The sample size was pragmatically determined based on the typical patient admission rates to the General Medicine ward and the availability of clinical staff for participation. The goal was to generate preliminary data on feasibility, acceptability and potential impacts of embedding a PCNC to inform future, larger‐scale evaluations.

### 3.2 Planning the Intervention

During the six‐week study, a PCNC was embedded within the General Medical Ward. The PCNC's primary role included care coordination, acting as a family liaison and providing follow‐up services for study participants. This involved facilitating discussions with families, medical staff, nursing teams and allied health professionals, as well as participating in both informal and formal family meetings. Input was required to optimise symptom management, with common issues such as constipation, pain and dyspnoea also being managed by the general medicine team.

Each morning, the PCNC met with General Medicine teams to identify eligible patients and attended daily multidisciplinary meetings, involving medical teams, senior nurses and allied health staff (including social workers, physiotherapists, occupational therapists, dieticians and speech pathologists). The PCNC also participated in ‘Going Home Rounds’ to address the needs of complex patients with extended hospital stays and discharge barriers, recognising that such stays often indicated poor prognostic outcomes.

During the intervention, researchers did not manipulate or randomise participants. Observations focused on the integration of the PCNC into ward routines, referral patterns and interdisciplinary care processes. The PCNC served a dual role as both practitioner and observer, maintaining detailed field notes during patient reviews, family discussions and interdisciplinary handovers. Observations were qualitative and documented daily to capture emerging themes around role feasibility, communication patterns and care outcomes. No structured observational tools were used; rather, reflective notes and clinical logs formed the basis of insight generation. The PCNC did not interact with or influence patient care directly, which was beyond the standard scope of the PCNC role.

### 3.3 Data Collection

Patients meeting the inclusion criteria were approached by the PCNC, who also engaged with family members upon obtaining consent. For patients with cognitive impairment or acute illness, the next of kin was contacted. Discussions focused on patients understanding of their current medical issues, assessing quality of life and exploring future treatment preferences, including the potential appointment of a medical treatment decision maker (MTDM) and/or completion of an ACP. Observational data were collected prospectively by the PCNC through daily clinical logs, reflective field notes and tracking of referral patterns and care processes. Each patient's care trajectory, including time of referral, location of care, goals of care discussions and discharge outcome, was documented in real‐time using a standardised template. Notes were reviewed weekly by the research team to identify patterns related to timing, acceptability and feasibility of PCNC involvement.

To explore allied health and nursing health care professionals' beliefs and practices in palliative care, a survey was developed addressing: (1) level of confidence in identifying patients with palliative care needs, (2) level of comfort discussing palliative care needs with medical staff, (3) factors influencing timely referrals, (4) barriers to advocating for palliative care and (5) perceived benefits of integrating a palliative care nurse into the general medicine team.

### 3.4 Data Analysis

Quantitative data were entered into a Microsoft Excel database and analysed by the study investigators. Descriptive statistics, including numbers, ranges and percentages, were used to summarise participants' demographic information. Qualitative data were gathered from two sources: (1) field notes and clinical reflections recorded by the PCNC during observation activities and (2) structured feedback from nursing and allied health staff involved in patient care. Qualitative data from the survey were analysed thematically to identify key patterns and provide meaningful insights. To enhance trustworthiness, an independent co‐investigator reviewed a subset of the data and preliminary codes. Discrepancies were resolved through discussion until consensus was reached.

Staff feedback was gathered via a brief, structured survey comprising closed‐ and open‐ended questions on experiences with the PCNC role, its impact on care, and perceived enablers and barriers. Due to the study's exploratory nature and small sample size, formal validation and reliability testing (e.g., Cronbach's alpha) of this survey were not conducted. Nevertheless, the questionnaire, developed from relevant literature and clinical priorities, was peer‐reviewed by three palliative care experts and piloted with two staff members for clarity.

## 4 Ethical Considerations

Ethical approval for this study was obtained from the Monash Health Human Research Ethics Committees (approval No: QA/100504/MonH‐2023‐384915(v1)), with approval granted on 21 July 2023. Informed consent was obtained from all participants or, where applicable, from their legally authorised representatives prior to participation. Participants were assured of the confidentiality of their data and the voluntary nature of their involvement. All data were collected, stored and managed in compliance with institutional privacy protocols and relevant data governance frameworks. Some patients in this study experienced cognitive impairment due to advanced illness or comorbidities, which may have impacted their capacity to provide informed consent. In such cases, a surrogate decision‐maker (e.g., family member or legally appointed guardian) was approached to provide consent on the patient's behalf. The research team also employed a brief cognitive screening and clinical judgement to assess consent capacity and verbal assent was sought from patients wherever possible, even when surrogate consent was used.

Given the cultural and linguistic diversity of participants, standard interpreter protocols as per Monash Health policy were strictly followed. Accredited professional interpreters were used during patient recruitment, consent discussions and follow‐up communications whenever language barriers were identified. Where appropriate, family members were consulted in addition to, but not as a replacement for, professional interpreters to support cultural and emotional context. Translated materials, including patient information sheets and consent forms, were provided in the participant's preferred language where available. During key stages of the study: recruitment, informed consent, data collection and follow‐up, language support was consistently integrated to ensure comprehension and participant comfort. The PCNC adopted a person‐centred, culturally sensitive communication style, including adapting pace, tone and discussion format, to align with participants' values and preferences. These measures ensured ethical engagement with CALD participants and promoted inclusive research practice.

## 5 Results

### 5.1 Patient Demographics

A total of 20 patients were recruited for the study (Table 1), aged from 67 to 97 years, with an average age of 87 years. The average number of hospital admissions in the prior six months was 1.8, ranging from 1 to 6. The average hospital length of stay during those admissions was 23.7 days, spanning 3–88 days. Of those patients admitted to the Palliative Care Unit (PCU) prior to death, the average duration of stay was 3 days, ranging from less than 24 h (2 patients) to 8 days. Regarding preferred language, 55% of the 20 patients spoke a language other than English and 65% were born outside of Australia. Detailed demographic data for all participants are presented in Table 1.

**TABLE 1:** Demographic information of the patients (*N* = 20).

Demographic characteristic	Category	*n* (%)
Living circumstances	Home alone	4 (20%)
	Home with family	12 (60%)
	RACF	4 (20%)
Country of birth	Australia	7 (35%)
	Greece	3 (15%)
	Vietnam	3 (15%)
	UK	2 (10%)
	Other	5 (25%)
Preferred language	English	9 (45%)
	Language other than English	11 (55%)
Religion	Christian	7 (35%)
	Greek Orthodox	3 (15%)
	Russian Orthodox	1 (5%)
	Buddhist	2 (10%)
	Islam	1 (5%)
	Unknown	6 (30%)
Decision making capacity	Full	4 (20%)
	Some	7 (35%)
	Nil/limited	9 (45%)
ACP/Medical treatment decision maker/treatment preferences discussed with patient and or family	Patient only	3 (15%)
	Family only	13 (65%)
	Patient and family	4 (20%)
	Not discussed	1 (5%)
	Medical treatment decision maker (MTDM) appointed	3 (15%)
	ACP	0 (0%)
PCNC interventions (More than 1 intervention for each patient)	Care coordination	8 (40%)
	Family liaison	15 (75%)
	Symptom management	10 (50%)
	Follow up services	3 (15%)
Discharge outcome	Discharged home	4 (20%)
	Sub‐acute	6 (30%)
	PCU	4 (20%)
	RACF	2 (10%)
	Deceased in acute care	4 (20%)

Abbreviations: ACP, advance care planning; MTDM, medical treatment decision maker; PCNC, palliative care nurse consultant; PCU, palliative care unit; RACF, residential aged care facility.

### 5.2 Discharge Outcome

**FIGURE 1:**
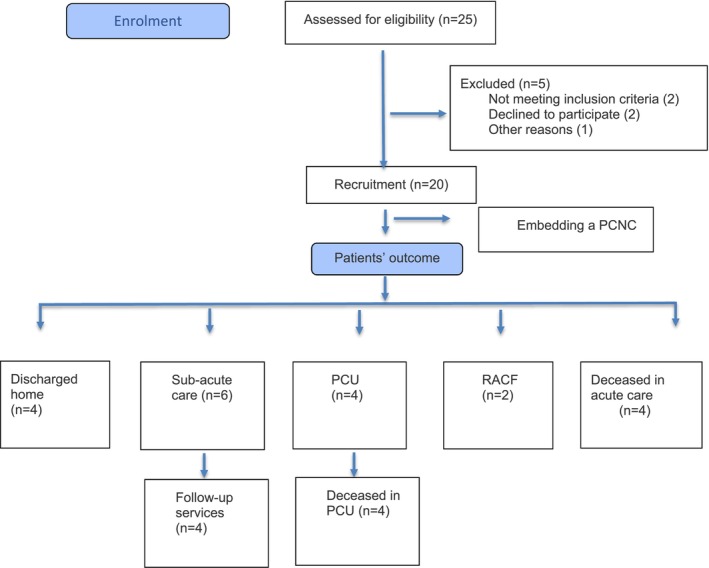
Flow diagram for patient recruitment and outcomes. PCNC, palliative care nurse consultant; PCU, palliative care unit; RACF, residential aged care facility. [Colour figure can be viewed at wileyonlinelibrary.com]

The study identified four discharge outcomes among the 20 participants: 4 were discharged home, 6 transferred to sub‐acute care, 4 to a PCU, 2 to residential aged care facilities (RACF), whilst 4 died in acute care. Among those transferred to sub‐acute care, 4 received follow‐up services, including referrals to a Supportive Care Clinic (SCC), Community Palliative Care (CPC) and the Palliative Care Consultancy Service (PCCS). See Figure 1 flowchart for patients' outcome.

Notably, eight patients died during the study, with four in acute care and four in the PCU. Three families declined PCU transfer. Barriers to PCU transfers included patients and/or their families not wanting the patient disturbed given their limited life expectancy, concerns regarding ambulance transfer to PCU (located on the same hospital campus but in an external building requiring ambulance transfer) and changing their care team with whom they had developed a relationship through difficult discussions. Another patient was too unstable at the time the referral to the PCCS was made. Of the four patients transferred to the PCU, 2 patients died in less than 24 h, a further patient after two days, and another after eight days post transfer.

### 5.3 Decision Making Capacity and Treatment Preference

In this study, only four patients retained full decision‐making capacity. The remaining 16 had limited or no capacity due to acute illness, persistent delirium, or pre‐existing cognitive impairment. Consequently, most discussions were conducted with family members, who universally acknowledged the patient's increasing frailty and the importance of future planning. Three patients had a MTDM appointed, and one had completed an ACP. These were pre‐existing MTDM completions, and there were not any discussions about MTDM or ACP.

### 5.4 Post Study Patients and Family Follow Up and Feedback

It was recognised that patient and family feedback would be important in demonstrating the effectiveness of the project. However, due to the small scale of the study, there was limited capacity to undertake this. Furthermore, the cohort was not as anticipated, with a high proportion of terminal care patients and individuals with impaired cognition. Contact was successfully made with five patients or their family members, while follow‐up was abandoned after three unsuccessful attempts for others. A common theme that emerged across these five follow‐ups was the *importance of early engagement enabling home‐based end‐of‐life care*. Families valued the role of the PCNC in initiating sensitive conversations early in the admission, which allowed preferences such as dying at home to be recognised and honoured. This highlights the potential value of embedding PCNCs in facilitating care aligned with patient and family wishes.

One patient, after an eight‐week hospital stay, was supported to remain at home through early discussions with the PCNC and accepted a referral to community palliative care services when deterioration occurred. He remained out of hospital and died at home in accordance with his wishes. Another patient, who had previously described herself as ‘not being a quitter’, experienced an acute deterioration and her granddaughter, informed by earlier PCNC discussions, was receptive to a timely referral to the Palliative Care Consultancy Service. These cases highlight that introducing palliative care conversations to potential patients during more stable phases can enhance acceptance and planning during subsequent acute deteriorations.

### 5.5 Demographics of Healthcare Professionals

Of the 13 nurses who completed the survey, most were Grade 2/Year 3 or higher, employed in roles such as Clinical Support Nurse, Clinical Nurse Specialist, Associate Nursing Manager, Nursing Manager and Nurse Educator. Of the 5 allied health staff who participated, 2 were Grade 1, 2 were Grade 2, and 1 was Grade 3 (Table 2). This included social workers, physiotherapists and dietitian healthcare professionals. See Table 3 for representative healthcare professionals' quotes by theme.

**TABLE 2:** Level of confidence, comfort and barriers as reported by clinicians within the survey.

		Nurses (*N* = 13)	Allied health (*N* = 5)
How confident do you feel in identifying patients who would benefit from palliative care involvement	Not confident	0	0
	Somewhat confident	2 (15%)	2 (40%)
	Confident	5 (39%)	1 (20%)
	Very confident	6 (46%)	2 (40%)
How comfortable do you feel discussing patients with medical staff, who you believe would benefit from palliative care involvement	Not comfortable	1 (7%)	0
	Somewhat comfortable	2 (15%)	2 (40%)
	Comfortable	5 (39%)	2 (40%)
	Very comfortable	5 (39%)	1 (20%)
How often do you experience barriers to advocating for palliative care referrals when you think you might be helpful for the patient and/or family members	Very often	1 (7%)	0 (%)
	Often	4 (32%)	2 (40%)
	Sometimes	5 (39%)	2 (40%)
	Not very often	2 (15%)	0 (%)
	Never	1 (7%)	0 (%)
	Missing response		1 (20%)
Do you believe there were any benefits in having a palliative care nursing working with the general medicine team during the 4 week project	Yes	11 (100%)	5 (100%)
	No	0	0

**TABLE 3:** Representative participant quotes by theme.

Theme	Representative quote
Confidence identifying patients	‘I know when someone would benefit from palliative care, but I don't always feel it's my place to raise it with the doctors.’—Nurse
Comfort discussing with doctors	‘I feel confident identifying the need, but less sure how to bring it up without overstepping.’—Allied Health Staff
Perceived benefits of PCNC	‘The PCNC was able to ask difficult questions of medical staff regarding unstable patients more quickly than allied health professionals.’—Allied Health Staff
Enhanced family communication	‘PCNC was helpful in answering questions when hard news was being delivered.’—Nurse
Barriers to advocacy	‘It's hard to bring up palliative care when the team is focused on discharge planning—it's seen as a last resort.’—Nurse
Cultural challenges	‘Patients and family members were reluctant to engage with palliative care due to limited information regarding prognosis.’—Nurse
Training needs	‘We need more knowledge and education on how to frame these conversations—it's not always clear where our role starts and ends.’—Allied health staff

Abbreviation: PCNC, palliative care nurse consultant.

### 5.6 Levels of Confidence and Comfort

Referrals to palliative care are usually made by the medical team and require (1) recognition of palliative care indications as well as (2) confidence to have this discussion with patients and/or their MTDM.

Senior nurses demonstrated higher confidence in identifying patients suitable for palliative care involvement, with 90% indicating they were ‘confident to very confident’. Similarly, 90% of nurses felt ‘comfortable to very comfortable’ discussing patients' palliative care needs with medical staff. However, they did not feel confident communicating their observations about readiness for palliative care to the doctor. One nurse explained, ‘I know when someone would benefit from palliative care, but I don't always feel it's my place to raise it with the doctors.’

In contrast, the most senior allied health staff reported being ‘very confident’ in identifying patients for palliative care and discussing this with medical staff, while Grade 1 and 2 respondents expressed ‘somewhat confident/confident’ levels. Likewise, 80% of allied health staff reported ‘somewhat comfortable to comfortable’ discussing palliative care needs with medical doctors. As one allied health team member stated, ‘I feel confident identifying the need, but less sure how to bring it up without overstepping.’

### 5.7 Perceived Benefits

All nurses affirmed that embedding PCNCs within general medicine wards provided significant benefits for patients and the treating teams, including enhancing medical teams' understanding of palliative care, fostering earlier discussions, improving medication management and empowering nurses to advocate for timely referrals. PCNCs also contributed to improved patient‐centred care, communication and timely referrals. Similarly, all allied health staff reported that PCNCs facilitated critical discussions, clarified clinical trajectories and reduced the stigma associated with palliative care, thereby empowering allied health teams to address end‐of‐life needs more effectively. One nurse noted, ‘PCNC was helpful in answering questions when “hard news” was being delivered.’ Another Allied health staff shared, ‘The PCNC was able to ask “difficult questions” of medical staff regarding unstable patients more quickly than allied health professionals.’

### 5.8 Perceived Barriers

Barriers to advocating for palliative care needs of patients were reported by 80% of nurses. Common barriers included delayed discussions about Advanced Care Directives (ACDs), prioritisation of patient flow constraints medical team's capacity of future care planning, reluctance of family members to address end‐of‐life care, delays in medical referral to palliative care, and stigma around palliative care. One nurse commented, ‘It's hard to bring up palliative care when the team is focused on discharge planning, it's seen as a last resort.’ Nurses also noted resistance from families and medical teams to refer to palliative services, with noticeable gaps in understanding about the role and scope of what palliative care could offer. *Another nurse added*: ‘Patients and family members reluctance to engage with palliative care due to limited information regarding prognosis.’ In Allied health respondents, 80% reported encountering barriers to advocating for palliative care. These were described as challenges with rotating medical teams, the fluctuating strength of the relationship between the medical and allied health team, unclear patient trajectories, resistance from families and inadequate palliative care training. As one allied health professional noted ‘We need more knowledge and education on how to frame these conversations, it's not always clear where our role starts and ends.’

## 6 Discussion

Our findings demonstrated referrals to palliative care services can be delayed for several reasons: (1) unpredictable prognosis and trajectories, particularly for non‐malignant disease; (2) prioritisation of patient flow that limits the medical team's capacity to engage in future care planning; (3) variable levels of confidence and experience with prognostication and discussions regarding treatment limitations in clinicians providing care in general medicine wards. Together, these factors hindered timely care, potentially resulting in unnecessary healthcare costs and societal burden (Bodenheimer and Sinsky 2014; Boscolo et al. 2020; Iglesia et al. 2020).

The aim of the study was to engage with patients earlier in their disease trajectory to explore their understanding of their medical condition and future treatment wishes whilst they still had capacity to contribute to these discussions. The findings highlighted some important issues that are relevant to the care of patients with complex and advanced disease in an acute care setting, as well as the challenges faced by the health professionals that care for them. While some disease‐specific indicators for chronic disease such as chronic respiratory and heart failure exist, they are imperfect in terms of predicting disease progression and pathways of care in palliative stages (Gebresillassie et al. 2024; Ng et al. 2024). This small cohort highlighted the unpredictable illness trajectory in the patients admitted to a General Medicine ward. Only one patient was admitted with a discreet diagnosis of congestive cardiac failure (CCF). The majority of patients were admitted with a specific event or tipping point such as an infection or a fall, and their progress followed various trajectories compounded by their co‐morbidities.

Initially, the 3‐month ‘surprise question’ (Lakin et al. 2016; Romo and Lynn 2017) was chosen as a simple trigger to identify suitable patients. For example, ‘*would you be surprised if this patient died in the next 12 months?*’, which is a validated prognostic tool that supports early recognition of patients with limited life expectancy. However, it is likely that the ‘surprise question’ itself is too vague. Ultimately, using this trigger question, referrals were quite late, with patients dying quickly on admission to the PCU and many patients not having decision‐making capacity to participate on referral. The result was in line with a systematic review and meta‐analysis study conducted by Downar et al. (2017) who stated that the 3‐month ‘surprise question’ demonstrated worse performance in noncancer illness as a predictive tool for death.

Daily multidisciplinary team meetings were anticipated to be the primary forum for identifying suitable patients; however, these meetings were highly time constrained, with approximately 50 patients discussed within a 40‐min timeframe. The primary focus of these discussions centred on determining the need for allied health involvement and finalising discharge plans.

The primary intervention of the PCNC was care coordination and family liaison. When feasible, PCNC also attended formal family meetings to discuss limitations of treatment for deteriorating patients and explore discharge planning. These meetings, though resource‐intensive for medical teams, were more efficient than daily bedside updates, especially for patients with poor recall or cognitive impairments. The PCNC's presence, as evidenced in patients and/or family feedback, was critical in facilitating sensitive discussions, advocating for patient wishes, and addressing end‐of‐life care decisions. In addition, PCNC was able to build the bridge between the treating team and palliative care in what was seen by family members as a less confronting transition. The prospective design of PCNC involvement enabled direct patient and family engagement, aligning with evidence from similar embedded palliative care models in intensive care settings (Mehta et al. 2023).

Our study identified four discharge outcomes, with a recommendation for PCNC's intervention focus in each category.

1. **Terminal phase (dying within hours to days)**: Opportunities for home discharge for end‐of‐life care were often missed, and several families preferred their loved ones not to be disturbed by a transfer to a PCU during their final hours or days.
2. **Pre‐terminal phase (dying within days to weeks)**: This cohort underscored the need for enhanced direct palliative involvement. Key areas of PCNC's intervention included supporting patients, families and healthcare professionals in navigating difficult discussions about withholding or withdrawing treatment and identifying the most appropriate care setting based on patient preferences.
3. **Transfers to sub‐acute care or RACFs**: Patients in this group frequently transitioned to sub‐acute settings, where their stabilised condition provided a better environment to explore values and preferences. High‐risk patients were flagged to PCCS for follow‐up. Routine discussions about care preferences were prioritised for patients awaiting RACF placement.
4. **Home discharges**: This group required post‐discharge follow‐up through appropriate services such as CPC, ACP and SCC, in coordination with General Practitioners. This study highlighted that despite a well‐established PCCS, negative perceptions of palliative care persist. While palliative care referrals often focus on symptom control, there is resistance to its broader role, particularly in cases without complex symptoms. The rebranding of palliative care as ‘supportive care’ or ‘plus care’ has been a topic of debate (Dai et al. 2017), with a transitional or bridging framework sometimes found to enhance the acceptability of palliative care among patients, families and healthcare providers.

The study found that none of the 20 patients instigated ACP. When prompting family members about the completion of ACP processes, a common response was that they had been too busy dealing with multiple issues related to discharge and had not been able to prioritise completing it. This study aligns with existing literature indicating that ACP uptake in this population is low, particularly among ethnic and racial minorities (González‐González et al. 2020; Harrison et al. 2016; Jimenez et al. 2018). Therefore, very limited information is known about their treatment preferences. Systematic reviews and meta‐analyses have reached a general consensus that advance care planning can positively impact the concordance between individual preference and care, quality of life and family burden (Khandelwal et al. 2015; Martin et al. 2016; Schichtel et al. 2020). In addition, hospitalisations, length of stay and ICU admissions can also be reduced (Johnson et al. 2018; Khandelwal et al. 2016). A recent integrative review demonstrated that enhanced communications via standardised structured or informal dialogue can increase the likelihood of patient and/or family engagement with ACP (Goswami 2023).

Timely referrals to palliative care services to meet the complex care needs of critically ill patients require the cooperation of multidisciplinary healthcare professionals (Hui et al. 2022; Mertens et al. 2021). Our study found that all healthcare professionals agreed on the benefits of having PCNC collaboration with the general medicine team, aligning with the findings of Gatta and Turnbull (2018). Bedside nurses expressed a lack of confidence in raising concerns about burdensome treatments for deteriorating patients when these were thought to be futile. It appeared that the cultural background of individual nurses influenced perceptions of futility, while time constraints, lack of continuity, large wards and a junior workforce hindered clinical confidence and advocacy. Functional status was assessed using the Palliative Performance Scale (PPS) (Anderson et al. 1996), a validated tool ranging from 0% (death) to 100% (full ambulation and normal activity), which helps to quantify patients' physical performance and guide care planning in palliative settings. It seems that using the PPS, in addition to the ‘surprise question’ led to greater engagement among nursing staff and enhanced their confidence in identifying suitable patients.

The limited number of follow‐up conversations (*n* = 5) was constraint of the study. This was largely due to the high proportion of patients with cognitive impairment or rapid clinical deterioration. Future studies should adopt more systematic approaches to capture family feedback, such as semi‐structured interviews or validated tools like the FAMCARE‐2 survey (Tonboot et al. 2025), to ensure a more comprehensive evaluation of palliative care experiences. These methods could help improve both response rates and depth of insight.

## 7 Limitations

It is acknowledged that this prospective quality assurance study has several limitations. The study included only 20 patients and 18 healthcare professionals, with medical doctors being omitted from the survey due to resource constraints. This may limit the generalizability of the findings to broader populations or settings. Additionally, the exclusion of medical doctors represents a gap, as it restricts the study's ability to fully examine institutional or clinician‐level barriers to timely referrals. Inclusion of this stakeholder group in future research is essential for a more comprehensive understanding of referral dynamics and interprofessional collaboration. As the study was conducted in a single General Medicine ward at Monash Medical Centre, the patient cohort, institutional culture and care processes at Monash Medical Centre may not reflect broader hospital environments or diverse healthcare systems. In addition, the six‐week study period will not have captured long‐term outcomes, such as the sustained impact of PCNC interventions on patient care, staff confidence, ACP uptake and healthcare utilisation after discharge (e.g., readmissions). Hence, Caution should be exercised in extrapolating these results to other settings without further validation. However, the study's setting, a large public metropolitan hospital with a culturally diverse patient population may reflect challenges common to other urban acute care environments, suggesting that the observed benefits and barriers could have relevance beyond the immediate context. Despite this, the study provides valuable insights regarding the feasibility of integrating palliative care within a general medicine ward.

An additional limitation relates to the culturally and linguistically diverse (CALD) nature of the study population. While accredited interpreters and translated materials were used where appropriate, challenges inherent to multilingual research remain. These include the risk of nuanced meanings being lost in translation, variability in interpreter communication styles and the difficulty of fully capturing emotional or culturally embedded expressions. Interpreter‐mediated communication may also influence the dynamics of sensitive conversations, particularly around end‐of‐life care. Hence, future research should consider incorporating multilingual research personnel and cultural liaison roles to further strengthen communication quality and contextual interpretation.

Several methodological limitations must be acknowledged. First, while this study employed both quantitative and qualitative methods, it was not designed as a formal mixed‐methods study, and integration of findings occurred at the interpretation rather than design stage. Second, the PCNC served a dual role as both interventionist and observer, which introduces a risk of observer bias; however, reflective field notes and oversight by the broader research team aimed to mitigate this. Third, the small patients sample size (*n* = 20) and absence of a formal power calculation limit generalisability. Patient identification relied on physician discretion, which may have introduced selection bias. Additionally, no formal qualitative interview protocols were employed, and family feedback was limited to five cases, restricting thematic depth.

The staff survey, while peer‐reviewed, was only pilot tested with two individuals, and qualitative data analysis did not employ software or formal intercoder reliability checks. Coding was conducted manually by one researcher and reviewed by a second for consensus. While thematic saturation was not formally evaluated, recurring themes emerged consistently Additionally, data triangulation was not achieved, as the study relied primarily on direct observation and staff self‐report via surveys, without Data S1 sources such as medical record audits or structured family interviews. Future research should incorporate multi‐source data triangulation to strengthen analytic rigour and reduce potential bias.

## 8 Recommendations and Implications

This study emphasises the critical role that a PCNC embedded in general medicine can have for patients with complex healthcare needs and their families, providing insights for clinical research, education, policy development and practice. Future studies should further explore barriers and explore and design optimal service delivery for general medicine patients with complex healthcare needs, particularly for patients with non‐malignant conditions. Future studies should prioritise capturing the perspectives of medical staff to better understand clinical decision‐making processes and address barriers to timely palliative care referral from a multidisciplinary perspective. The long‐term impact of embedded palliative care models on patient outcomes, healthcare utilisation and clinician experiences should also be a focus of future research. Longitudinal studies are recommended to assess whether PCNC involvement contributes to sustained benefits, including reduced hospital readmissions, increased ACP documentation post‐discharge and alignment of care with patient preferences in the months following hospitalisation.

Future research should also include larger, multi‐centre studies to assess the scalability and impact of this model across different institutional contexts, geographic regions and patient populations.

Structured training programs for generalist healthcare workers focusing on palliative care principles and communication skills are essential. These programs should address cultural competency, ethical decision‐making and strategies to navigate challenging conversations with patients, families and healthcare teams. For policy makers, the findings highlight the need for standardised referral criteria and improved resource allocation to support palliative care services. Policies should prioritise directly embedding a palliative care clinician in acute care settings.

In practice, embedding PCNCs within multidisciplinary teams fosters proactive discussions about treatment limitations and end‐of‐life care. Strengthening bedside nurses' confidence through tools like the Palliative Performance Scale (PPS) and regular ‘walk‐arounds’ with senior staff can enhance patient advocacy. Additionally, promoting ACP uptake through structured interventions and family engagement can improve alignment between care preferences and treatment outcomes. Embedding a PCNC within a general medicine ward appears feasible and acceptable within the study setting; however, broader implementation would require consideration of staffing capacity, funding models and institutional support. Key enablers include executive leadership buy‐in, integration with existing referral pathways and alignment with hospital quality and safety goals. Resource implications such as dedicated funding for a palliative care clinician, access to interdisciplinary collaboration are critical for sustainability. Future implementation efforts should evaluate cost‐effectiveness, workforce readiness and the adaptability of this model in diverse clinical contexts.

While embedding a PCNC within general medicine appears beneficial, alternative models warrant consideration for broader scalability. These include mobile interdisciplinary palliative care teams who provide bedside consults across wards, or peer ‘palliative care champions’ embedded within nursing or allied health staff who receive targeted training and mentorship. Such models may reduce resource burden and facilitate more sustained culture change. Future comparative studies should evaluate the effectiveness, feasibility and acceptability of these models across varied acute care contexts.

While this study was conducted in a large, urban tertiary hospital with access to specialist staff, implementing a PCNC model in under‐resourced settings may present significant challenges. Rural hospitals and health systems in low‐ and middle‐income countries often face workforce shortages, limited access to specialist palliative care and fewer institutional supports for interdisciplinary collaboration. In such contexts, alternative models such as training generalist nurses in core palliative care principles or developing telehealth‐enabled consults may be more feasible. Implementation research in these settings is urgently needed to assess how palliative care integration can be adapted effectively and equitably across diverse healthcare systems.

## 9 Conclusion

This study highlights the significant value of embedding a PCNC within general medicine units to address the complex needs of older patients with non‐malignant conditions. Despite barriers such as delayed referrals and variable clinician confidence, the PCNC involvement facilitated improved care coordination, proactive discussions and timely decision‐making. These findings emphasise the importance of integrating palliative care early in the patient trajectory, aligning care with individual preferences and supporting healthcare professionals through structured tools and education.

## Author Contributions

J.Z., C.M., O.H., G.V., R.M., M.D. and B.C. all contributed to the design of the study. C.M. collected and analysed the data. J.Z. and C.M. contributed to the overall writing up of the manuscript and all authors contributed to the revision of the manuscript. All authors read and approved the final manuscript.

## Ethics Statement

The study was approved by Monash Health Human Research Ethics Committees (approval No: QA/100504/MonH‐2023‐384915(v1)).

## Conflicts of Interest

The authors declare no conflicts of interest.

## Supporting information


**Data S1:** jocn70088‐sup‐0001‐DataS1.docx.

## Data Availability

The data that support the findings of this study are available from the corresponding author upon reasonable request. Restrictions apply to the availability of these data due to confidentiality reasons.
